# From the ground up: strengthening health systems at district level

**DOI:** 10.1186/1472-6963-13-S2-S2

**Published:** 2013-05-31

**Authors:** Mary T Bassett, Elaine K Gallin, Lola Adedokun, Cassiopeia Toner

**Affiliations:** 1 The Doris Duke Charitable Foundation, New York, NY, 10019, USA; 2 Dr. Gallin was the program director for the Medical Research Program at the time the African Health Initiative was launched. She currently works as an independent consultant

## Introduction

**Figure 1:**
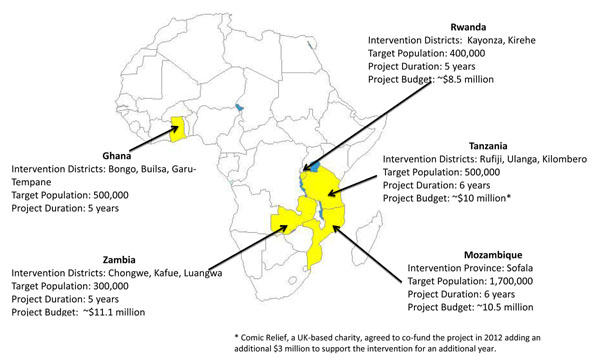
Population health implementation and training partnership sites

This supplement introduces the African Health Initiative (AHI), a research program comprised of five unique district health system-strengthening activities in Ghana, Mozambique, Rwanda, Tanzania, and Zambia that began in 2009 (Figure 1). This supplement should be of interest to all engaged in improving delivery of district primary health care — whether ministries of health, service providers, funders, or those who evaluate complex interventions. The five AHI projects, known as Population Health Implementation and Training (PHIT) Partnerships, are funded by the Doris Duke Charitable Foundation (DDCF) with a common goal: to produce significant, measureable health improvements in a defined geographic area over a five to seven year grant period. With the partnerships in their fourth year of funding, it is now possible to capture lessons learned in project design and implementation. Evaluation of the African Health Initiative’s impact on population health, including mortality, however, must await the conclusion of the grant period.

## Why focus on health systems?

The last two decades saw unprecedented growth in the level of assistance available for health in developing countries. Although health expenditure by African governments remains below the Abuja Declaration target (15% of government expenditure) [1], donor support for health more than tripled between 1990 and 2008 [2] , reaching more than $27 billion annual expenditure in 2010 [3]. Much of this support is directed at “big diseases”, notably HIV/AIDS, tuberculosis, and malaria, often under the auspices of new global health initiatives. These laudable, ambitious efforts to target major killers encountered already fragile, under-resourced health systems that limited the capacity of beneficiary countries to absorb new investments. Although there are no simple, fast solutions to strengthening health systems, the World Health Organization [4] and others, notably the Alliance for Health Policy and Systems Research, have been central in promoting a dialogue on how to address this critical issue [5].

In 2006, as global health funding was increasing, the Doris Duke Charitable Foundation Board of Trustees was considering one or two new initiatives, spurred by a desire both to celebrate its upcoming 10^th^ anniversary of grant making and a surge in returns on its endowment. The Foundation’s Medical Research Program supported mainly domestic clinical research. Only a small portion of DDCF’s grant making was directed to research on the treatment and care of AIDS patients in Africa and the construction of the Doris Duke Medical Research Institute in Durban, South Africa. It was these latter activities that brought DDCF staff to several sub-Saharan African countries. On an early visit, staff were struck by the presence of a new HIV clinic — stocked with needed medicines and supplies and staffed by a proud health worker –- while across the street was a district hospital —derelict, no supplies, crumbling infrastructure, few staff, and patients lying on the ground for lack of beds. Further visits confirmed that, as AIDS treatment rolled out, such contrasts were common throughout sub-Saharan Africa. How could it possibly make sense for a woman to attend this new HIV clinic, and then have to contend with a non-functioning community clinic and an under-resourced district hospital for pregnancy care, childhood immunizations, or management of malaria? Not only did it seem logical that health clinics should provide integrated primary care for an entire family, but also that those clinics be part of a *health system* that could deliver drugs and supplies on time, train workers, and, when required, refer patients for treatment of complicated cases. Although how to define, assess, and measure health systems continues to pose challenges [6], it was these real-world observations, consultation with a range of experts, and the belief that the Foundation should be willing to address critical gaps even if they were ‘hard and high risk,’ that propelled DDCF to invest in health systems strengthening. Staff were heartened by findings of the Tanzanian Essential Health Interventions Project (TEHIP) [7] and the Navrongo Experiment in northern Ghana [8], both of which suggested that health systems interventions could indeed result in meaningful population health gains in a relatively short period of time.

## The Doris Duke Charitable Foundation African Health Initiative

By 2007, the DDCF Board had approved $60 million for the African Health Initiative to support a small portfolio of diverse approaches to health systems strengthening over a period of five to seven years (until 2015). A request for proposals was released requiring that applicants target a geographic area of at least 250,000 people and demonstrate a measureable impact on population health, including mortality. Funded groups — the PHIT Partnerships —were to be comprised of a U.S. institution (those selected were all universities) and institutions based in the country of focus. Additionally, Partnerships were to have a history of collaborative work in the targeted communities and explicit support of the country’s ministry of health. The RFP did not specify the interventions, because DDCF staff reasoned that those “on the ground” were best placed to determine which approaches were appropriate. The response to the RFP alone — 137 letters of interest, when staff had anticipated about 50 — pointed to untapped interest in funding opportunities for health system strengthening. In July 2009, after a two-step, peer-reviewed selection process [9], five Partnerships were funded.

## Implementation research

Many effective strategies for management of primary care conditions have been developed and tested, but implementation at scale often has been disappointing. In order to help bridge the widely described “knowledge-action gap” and contribute to the emerging field of implementation research [10], DDCF staff was emphatic that the Partnerships rigorously evaluate their efforts and share information with the public in a timely manner. All Partnerships agreed to make data publicly available within two-and-a-half years of completion of field work. To assess the ultimate goal of reducing preventable deaths within a five-seven year grant period, all five Partnerships selected measures of mortality. For most, this was under-5 mortality; the Zambian Partnership will also track adult mortality. Finally, all of the Partnerships will collect the data needed to answer the question, “How much would it cost to replicate this intervention?” Some will conduct further economic analyses, to assess, for example, cost-effectiveness and the impact of efforts to strengthen the health system on the occurrence of catastrophic health expenditures.

## A portfolio of health systems strengthening projects

**Figure 2:**
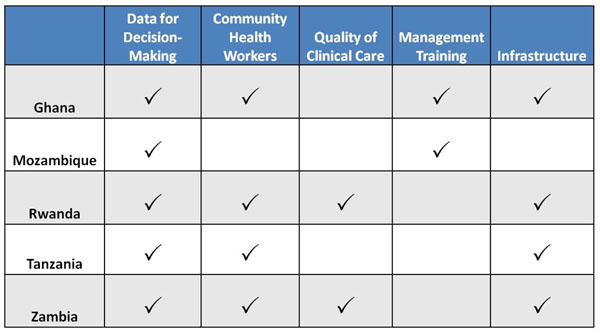
Matrix of intervention focuses

The Partnerships are geographically diverse and differ substantially in their design: each has its own intervention approach and unique evaluation strategies. Two Partnerships (Mozambique and Ghana) rely heavily on improving district management, two focus on strengthening a community-based cadre (Rwanda and Tanzania), and one on improving the quality of clinical management (Zambia). All of the projects became more expansive in scope as activities got under way. For example, the Zambian team hypothesized that high quality, protocol-driven, appropriately resourced care would result in better clinical outcomes and lower mortality. But, as implementation began, the team realized that improved health center based care would only have an impact if services were used and patients adhered to care. This led to a greater emphasis on community outreach workers than initially planned. Similarly, the Rwanda Partnership focused initially on expanding community based cadres and improving the infrastructure, material and human resource capacity of health centers. But, over the first years of the project, the team broadened its approach to include clinical mentoring, reasoning that expansion of resources alone would not assure quality improvement. All teams came to address, to varying degrees, the whole range of health system components as captured by the “six-building blocks” formulated by the World Health Organization — service delivery, health workforce, information, medicines, financing and governance [11]. All include use of health information to improve both clinical and management decisions. Most projects have evolved to include community-based activities to build demand and expand access to care, and health center based efforts to improve service quality and efforts to strengthen health systems management. All include use of health information to improve both clinical and management decisions (Figure 2).

## Managing program evolution and learning across projects

Each Partnership is implementing a set of dynamic, complex interventions that require a range of varied skills and occur within a changing landscape. With rollout, the iterative, adaptive nature of these interventions has become increasingly apparent. “Learning while doing” has been a *de facto* necessity, and, inevitably, what has happened on the ground has differed from what was planned on paper. This led to a growing recognition of the need to document events and activities in real-time, including the introduction of new interventions by other organizations (both government and non-governmental organizations) in Partnership areas. The challenges of evaluating a constantly evolving intervention are formidable. How will we know what actually has been done? Will the iterative process of improving practice, rather than the initial project design or specific strategies and interventions, be what actually determines success? How to describe and measure these processes in replicable, scientific ways is largely uncharted territory.

The very different approaches used by the five Partnerships also pose challenges to our aim of learning lessons across the entire initiative. Nonetheless, through a consultative process spearheaded by a “Data Coordinator” (described in [12]), the Partnerships agreed on measurement approaches that would maximize comparability, where possible. We have complemented this largely quantitative approach with more qualitative work designed to gain an understanding of the evolution and change processes in the five Partnership settings.

While the Partnerships have all drawn on the WHO six-building blocks approach to health systems strengthening, implementation has shown some limitations of this frame — an observation offered by others as well [6]. Dynamic, interactive elements of the system are not reflected in the six building blocks. In particular, the important role of communities in promoting their own health is not captured. The growing role of community health workers in primary health care delivery attests to increasing interest in assuring community engagement, but what constitutes a community health worker and to what degree community engagement includes community mobilization varies widely. While not designed to address this question, the interventions offer a range of strategies. Some community health workers undergo several months of training, others just a few weeks. The cadres are drawn varyingly from the communities they serve and have different levels of educational attainment. Their connection to the formal health sectors varies —some are volunteers, others are employees, others received compensation but are not salaried. In addition, whether households are approached singly or through a community mobilization process also varies. These variations offer a chance to reflect on how different approaches may have a bearing on implementation.

In bringing these experiences together, we hope to contribute to a growing understanding of what it means to work simultaneously to strengthen health systems and measure the impact of these efforts. Four years into this effort, it is clear that more of this type of work at district level is needed. It is in districts such as these that policies and programs become the practice that can save lives. For this reason alone, strengthening district health systems deserves more effort and more research.

## Competing interests

The authors declare that they have no competing interests.

## Declarations

This article has been published as part of *BMC Health Services Research* Volume 13 Supplement 2, 2013: Improving primary health care to achieve population impact: the African Health Initiative. The full contents of the supplement are available online at http://www.biomedcentral.com/bmchealthservres/supplements/13/S2. Publication of this supplement was supported by the African Health Initiative of the Doris Duke Charitable Foundation.
